# *TOLLIP* and *MUC5B* single nucleotide polymorphisms among interstitial lung disease patients from Western India

**DOI:** 10.3389/fmed.2026.1806788

**Published:** 2026-04-14

**Authors:** Tanya Athavale, Amita Athavale, Trisha Samant, Namrata Neman, Ridi Khatri, Pooja Jaiswal, Kunal Dabholkar, Somprakash Dhangar, Anshu Priya, Manisha Madkaikar, Vandana Pradhan

**Affiliations:** 1 Department of Pulmonary Medicine and Environmental Pollution Research Centre, Seth G S Medical College and King Edward Memorial Hospital, Mumbai, India; 2 Department of Clinical and Experimental Immunology, Indian Council of Medical Research, National Institute of Immunohaematology, Mumbai, India; 3 Department of Pediatric Immunology and Leukocyte Biology, ICMR - National Institute of Immunohaematology, Mumbai, India

**Keywords:** biomarkers, IFN-γ, ILD subtypes, *TOLLIP* haplotypes, *TOLLIP* SNPs

## Abstract

**Background:**

Genetic variants in *TOLLIP* and MUC5B influence innate immune signaling and mucosal defense and have been implicated in interstitial lung disease (ILD) susceptibility. However, data from Indian populations remain scarce. This study aimed to characterize single nucleotide polymorphisms (SNPs) in *TOLLIP* and *MUC5B* among patients with interstitial lung disease (ILD) and its subtypes. Additionally, it investigated the relationship between these genetic variants and inflammatory biomarkers, as well as the patterns of linkage disequilibrium (LD) in patients with ILD from Western India.

**Methods:**

This cross-sectional study enrolled 200 patients with ILD and 104 healthy controls. Six SNPs, *TOLLIP* rs3750920, rs111521887, rs5743890, rs5743894, rs5743854, and *MUC5B* rs35705950, were genotyped using Sanger sequencing and PCR-RFLP. Serum cytokine levels (IL-1β, TNF-*α*, IFN-*γ*, and IL-6) were measured using a multiplex bead-based immunoassay. LD was assessed using Haploview software.

**Results:**

Connective tissue disease-related ILD (CTD-ILD) was the most common subtype (45%). The *TOLLIP* rs3750920_T allele was significantly more frequent in idiopathic pulmonary fibrosis (IPF) than in controls (66.1% vs. 50.5%; *p* = 0.038). The *TOLLIP* rs111521887_G allele was more frequent in CTD-ILD than controls (18.4% vs. 7.4%; *p* = 0.002). The *TOLLIP* rs5743890_C allele was more frequent in controls than ILD (7.5% vs. 2.9%; *p* = 0.016). The *TOLLIP* rs5743894_C allele was more frequent in all patients with ILD than in controls (14.5% vs. 8.1%; *p* = 0.03), and specifically in the IPF subtype (19.2% vs. 8.1%; *p* = 0.01). The *TOLLIP* rs5743854_G allele was found to be more frequent in the idiopathic NSIP subtype as compared to controls (41.2% vs. 23.2%; *p* = 0.033). The *MUC5B* rs35705950_T allele was significantly more frequent in ILD than controls (19.6% vs. 5.8%; *p* = 0.0008). Among cytokines, IFN-γ was significantly elevated in *TOLLIP* rs5743854 C/C and *MUC5B* rs35705950 G/G homozygous genotypes in patients with ILD. Furthermore, LD analysis revealed no significant haplotype blocks in this population.

**Conclusion:**

This study provided the first population-specific data on *TOLLIP* and *MUC5B* genotypes in Indian patients with ILD, highlighting genotype-driven variation in IFN-γ and distinct allele-frequency patterns compared to Western cohorts. This study also indicated that one-third of the Indian patients with ILD, higher than the 6–7% from currently available Asian data, had genotypes that may benefit from NAC therapy.

## Introduction

Interstitial lung diseases (ILD) are a group of disorders with varied etiologies and pathologies that can lead to respiratory failure by impairing gas exchange in the alveoli. Dhooria et al. (1) estimated the incidence and prevalence of ILD in India to be 10.1–20.2 and 49.0–98.1 per 100,000 population, respectively. Our understanding of chronic respiratory diseases, including ILD, has evolved from recognizing the underlying cause to identifying genetic susceptibility to developing the disease.

Several single nucleotide polymorphisms (SNPs) within the *TOLLIP* locus were shown to be associated with idiopathic pulmonary fibrosis (IPF) (2). Since then, the association of SNPs within the *TOLLIP* locus has been found in asthma, chronic obstructive pulmonary disease (COPD), tuberculosis (TB), and Legionnaire’s disease (3). The Toll-interacting protein (*TOLLIP*) is an intracellular adaptor protein that functions as an inhibitor of inflammation and endoplasmic reticulum (ER) stress, an activator of autophagy, and a critical regulator of intracellular vacuole trafficking (3). Molecular regulation at the transcriptional and post-transcriptional levels explains the association of *TOLLIP* with pulmonary disease. The *TOLLIP* locus is hypermethylated in IPF, an example of epigenetic modification regulating gene expression (3). Different levels of regulation explain the variability in the association of minor alleles of *TOLLIP* SNPs with susceptibility to ILDs. Li et al. (4) included three *TOLLIP* variants—rs5743890, rs111521887, and rs3750920—in a meta-analysis. They found that rs5743890_C was associated with a lower incidence of ILD and worse survival in patients with IPF, whereas rs111521887_G and rs3750920_T were associated with an elevated risk of ILD but not with disease prognosis (4). In 2011, Seibold et al. fine-mapped a risk locus for idiopathic interstitial pneumonia (IIP) on the p-terminal of chromosome 11. They reported an association between the *MUC5B* SNP rs35705950 in the promoter region of the *MUC5B* gene and familial pulmonary fibrosis, as well as in IPF patients (5). The *MUC5B* rs35705950 promoter polymorphism is associated with IPF, interstitial lung abnormalities (ILA), interstitial lung changes after coronavirus disease of 2019 (COVID-19), connective tissue disease-related ILD (CTD-ILD), fibrotic hypersensitivity pneumonitis (fibrotic HP), and idiopathic non-specific interstitial pneumonia (idiopathic NSIP). The *MUC5B* rs35705950_T minor allele is associated with slower progression of IPF (6–10). In the PANTHER-IPF trial, Oldham et al. (11) reported that IPF patients with the *TOLLIP* rs3750920 T/T genotype could benefit from N-acetylcysteine (NAC) treatment, while those with the C/C genotype could be harmed (12). This established one of the first hypotheses of drug-gene interaction in treating ILDs. The pharmacogenomic approach is currently being investigated in phase 3 of the PRECISIONS trial, which will compare NAC versus placebo in patients with IPF who have a *TOLLIP* rs3750920 T/T genotype (11, 12). A pilot study by Kubbara et al. (13) also suggested that in IPF patients, the *TOLLIP* rs5743890 genotypes C/C and C/T may be associated with increased survival among patients treated with pirfenidone. *TOLLIP* and *MUC5B* are located in proximity on chromosome 11p15.5. Linkage disequilibrium (LD) studies of the two loci suggested that *MUC5B* rs3570950 and three neighboring *TOLLIP* variants (*MUC5B*-T920-T887-T894 haplotype block) formed haplotypes that were linked to increased susceptibility (T–T-C-T, T–T-G-C, T-C-C-T, G-C-C-T, and G-T-C-T) to fibrotic HP and identified a haplotype that decreased survival (G-T-G-C) in fibrotic HP patients (14). The *TOLLIP* rs5743890_C minor allele was more frequent in controls than in patients with systemic sclerosis (SSc), and the T/C haplotype was associated with an increased risk of progression in patients with SSc-ILD as reported by Schroder et al (15). Genetic variants in *TOLLIP* and *MUC5B* have been increasingly recognized as modulators of innate immune signaling and mucosal defense in ILD. Loss-of-function variants in *TOLLIP* (notably rs5743899 and rs3750920) have been associated with heightened TLR-mediated inflammation and increased susceptibility to IPF, suggesting that individuals carrying these SNPs may exhibit dysregulated cytokine responses. Elevated levels of IL-17A and IL-22 implicate Th17-mediated inflammation in ILD progression, while IFN-*γ* reflects Th1 polarization. IL-4 indicates Th2-driven fibrotic remodeling. Pro-inflammatory cytokines such as IL-1β, TNF-*α*, and IL-6 further amplify the fibro-inflammatory milieu characteristic of progressive ILD.

Cytokine profile in ILD may vary due to differences in genetic background, environmental exposures, and disease phenotype distribution (11, 16). Despite the growing incidence and prevalence of ILD in India and the pharmacogenomic relevance of its treatment, there is no data available on *TOLLIP* genotypes in Indian ILD subtypes. Studies on *TOLLIP* and *MUC5B* genotypes related to cytokine signatures in Indian patients with ILD are also lacking. This cross-sectional study aimed to bridge this gap by characterizing the profiles of *TOLLIP* and *MUC5B* single nucleotide polymorphisms (SNPs) in individuals diagnosed with interstitial lung disease (ILD) and its clinical subtypes. It further examined how these genetic variants relate to inflammatory biomarkers and explored linkage disequilibrium (LD) patterns among patients with ILD from Western India.

## Methods

### Enrolment of study participants

This cross-sectional study was conducted from May 2022 to May 2025. Ethics approval was obtained from the Institutional Ethics Committees of participating centers, *viz*., the Indian Council of Medical Research-National Institute of Immunohematology (ICMR-NIIH) (Project no: ICMR/NIIH-IEC/08/2021), Mumbai, India, and King Edward Memorial Hospital (KEMH) (Project no: EC/GOVT-05/2023), Mumbai, India. Patients with ILD (*n* = 200) aged over 18 years were recruited based on clinico-radiological evaluation with high-resolution computed tomography (HRCT) scans. Patients with ILD with clinical suspicion of any active infection, including COVID, tuberculosis, and neoplasm, were excluded. Subsequently, patients with ILD were subclassified in accordance with the guidelines and multidisciplinary discussion (MDD) (17–19). Additional procedures, including bronchoscopy and lung biopsy, were done as required to establish a diagnosis. After obtaining informed consent, detailed information on presenting symptoms, comorbidities, occupational and exposure history, and radiological findings was recorded.

Healthy controls (*n* = 104) were enrolled in this study. Molecular studies were performed on all 104 healthy individuals, and a serological workup was done in 51 healthy individuals due to limited resources. Healthy controls above 18 years of age were recruited from the referral unit of the Department of Clinical & Experimental Immunology, ICMR-NIIH, Mumbai, India, after screening for exclusion of individuals with breathlessness, chronic dry cough, any history of lung or rheumatic disease, active infections, tuberculosis, neoplasms, or cigarette smoking within the five years preceding their recruitment.

### Identification of single nucleotide polymorphisms (SNPs)

Peripheral whole blood samples were collected in EDTA vacutainers. Genomic DNA was extracted using the Qiagen DNA Mini Kit (Qiagen, Germany). DNA concentration and purity were assessed using a Qubit 4.0 fluorometer (ThermoFisher Scientific), and integrity was confirmed via 1% (w/v) agarose gel electrophoresis stained with ethidium bromide (EtBr). All primers were designed using Primer3 and validated for specificity using BLAST (20, 21). SNPs including *MUC5B* rs35705950, *TOLLIP* rs111521887, *TOLLIP* rs5743854, and *TOLLIP* rs5743890 were identified using Sanger’s sequencing method, amplified using locus-specific primers, and sequenced using the ABI 3730xl DNA analyzer (Applied Biosystems, USA). Sequencing results were analyzed using Chromas version 2.5.0 software. *TOLLIP* rs3750920 and *TOLLIP* rs5743894 SNPs were genotyped using the polymerase chain reaction-restriction fragment length polymorphism (PCR-RFLP) method. Amplified products were digested with the restriction enzymes MspI and FspI (New England Biolabs, USA), respectively. Digested fragments were resolved on 2.5–3% agarose gel to determine genotype patterns.

### Haplotype identification and linkage disequilibrium (LD)

The LD between *TOLLIP* and *MUC5B* gene SNPs was analyzed using Haploview version 4.1 software (22). The squared Pearson’s correlation coefficient (R^2^) and D prime (D′) were used as a measure of LD between pairs of SNPs using Haploview.

### Autoantibody profile and serum biomarker estimation

Blood collected in a plain vacutainer was centrifuged at 2000 rpm for 15 min to collect the serum. The serum was used to test for autoantibodies in all patients with ILD. An indirect immunofluorescence assay (IFA) for autoimmune workup was performed using HEp-2 cells and *Crithidia lucillae* to detect anti-nuclear antibodies (ANA) and anti-dsDNA autoantibodies, respectively (EUROIMMUN, Germany). Anti-neutrophil cytoplasmic antibodies (ANCA) were tested by IFA (EUROIMMUN, Germany). Serum levels of pro-inflammatory cytokines IL-1β (pg/ml), TNF-α (pg/ml), IFN-*γ* (pg/ml), and IL-6 (pg/ml) were estimated using multiplex bead-based immunoassay [AimPlex Biosciences, USA], acquired using BD FACS Diva™ Software v8.0.1 and analyzed using BD FCAP Array™ Software v3.0 [BD Biosciences, USA].

### Statistical methods

Statistical analyses were conducted using R Studio version 4.3.1 and GraphPad Prism version 10.0.0. The normality of data distribution was assessed using the Shapiro–Wilk normality test. Categorical variables were expressed in counts with percentages. Quantitative variables were expressed as median with the 25th to 75th percentile range as the interquartile range (IQR) for skewed distributions and as mean ± standard deviation (SD) for normal distributions. Mann–Whitney U and Kruskal-Wallis tests were used for skewed distributions. Student’s t-test and analysis of variance (ANOVA) were used for normal data to compare continuous variables. A two-tailed *p*-value less than 0.05 was considered statistically significant. While analyzing SNPs to test variant associations with ILD disease risk, the Hardy–Weinberg equilibrium (HWE) was assessed using the chi-square, and Fisher’s exact test was used in ILD and healthy controls. Allele frequencies in the healthy control population for all SNPs were calculated using South Asian data from the 1,000 Genomes database (23, 24).

## Results

### Clinical and radiological characteristics

The demographic, clinical, and radiological details of ILD patients (*n* = 200) are presented in Table 1. The men-to-women ratio was 1:2.2. Age- and sex-matched healthy controls (*n* = 104) were also enrolled. The present study reported the highest proportion of CTD-ILD (45% of patients, 90), as the patient enrollment was from a single tertiary center where pulmonologists, rheumatologists, radiologists, pathologists, and an autoimmune research laboratory were on the same premises. This setting facilitated a more efficient multidisciplinary evaluation, reducing the likelihood of missing diagnoses of CTD-ILD cases. This was followed by fibrotic HP (30 patients, 15%), IPF (30 patients, 15%), ‘other ILDs’ (28 patients, 14%), and idiopathic NSIP (22 patients, 11%). Among CTD-ILDs, 30 patients (33.3%) had rheumatoid arthritis-related ILD (RA-ILD), 12 patients (13.3%) had systemic sclerosis-related ILD (SSc-ILD), 8 patients (8.8%) had mixed connective tissue disorder-related ILD (MCTD-ILD), 3 patients (3.3%) had Sjogren’s syndrome- associated ILD (SjS-ILD), 2 patients (2.2%) had Systemic Lupus Erythematosus-associated ILD (SLE-ILD), 1 patient (1.1%) had myositis-associated ILD, and 1 patient (1.1%) had interstitial pneumonia with autoimmune features (IPAF). The remaining patients were categorized as ‘unclassified CTD-ILD’ (33 patients, 36.6%). Amongst the ‘other ILDs’ category, 6 patients (21.4%) had non-fibrotic HP, 3 patients (10.7%) had chronic pulmonary sarcoidosis, 7 patients (25%) had combined fibrosis with emphysema (CPFE), 4 patients (14.2%) had occupational ILD (*n* = 3 asbestosis; *n* = 1 silicosis), 2 patients (7.1%) had cystic lung disease, 1 patient (3.5%) had pulmonary alveolar proteinosis (PAP), 1 patient (3.5%) had pulmonary alveolar microlithiasis (PAM), and 4 patients (14.2%) had pulmonary fibrosis following acute respiratory distress syndrome (*n* = 1 after miliary tuberculosis and *n* = 3 after COVID-19).

**Table 1:** Baseline characteristics of study population (*n* = 200).

Baseline characteristics	All ILDs (*n* = 200)	CTD- ILD (*n* = 90)	Fibrotic HP (*n* = 30)	Idiopathic NSIP (*n* = 22)	IPF (*n* = 30)	Other ILDs (*n* = 28)	*p*-value
Age, years, mean ± SD	52.49 ± 15.08	47.53 ± 14.32	53.73 ± 15.18	56.77 ± 11.50	66.97 ± 7.632	48.11 ± 15.07	<0.001***^,@^
Women, *n* (%)	137 (68.5)	72 (80)	23 (76.6)	16 (72.7)	13 (43.3)	13 (46.4)	<0.001***^,$^
BMI, kg/m^2^, mean ± SD	23.73 ± 5.204	23.59 ± 6.124	23.83 ± 4.315	26.70 ± 3.196	24.03 ± 4.550	21.89 ± 4.583	0.134^@^
Smoking ever, *n* (%)	10 (5)	2 (2.2)	1 (3.3)	2 (9)	2 (6.6)	3 (10.7)	0.187+
Wood smoke exposure, *n* (%)	14 (7)	4 (4.4)	5 (16.6)	1 (4.5)	3 (10)	1 (3.5)	0.184+
Occupational exposure, *n* (%)	31 (15.5)	7 (7.7)	10 (33.3)	5 (22.7)	7 (23.3)	2 (9)	0.004**^,$^
Presence of comorbidities, *n* (%)	98 (49)	35 (38.8)	17 (56.6)	13 (59)	21 (70)	12 (42.8)	0.020*^,$^
FVC, percent predicted, mean ± SD	57.30 ± 19.54	60.24 ± 22.12	49.08 ± 16.26	49.38 ± 12.74	64.70 ± 17.53	57.20 ± 18.03	0.030*^,@^
Cough, *n* (%)	118 (59)	46 (51.1)	19 (63.3)	15 (68.1)	20 (66.6)	18 (64.3)	0.379$
Breathlessness, *n* (%)	150 (75)	62 (68.9)	24 (80)	14 (63.6)	24 (80)	26 (93)	0.052$
mMRC grade, mode	2	2	2	2	1	2	NA
Duration of symptoms (in months), Median (Q1-Q3)	12 (11–12)	12 (10–24)	12 (6–24)	24 (6–36)	7.5 (4–12)	12 (4–36)	0.053#
HRCT pattern							
UIP, *n* (%)	86 (43)	35 (38.8)	8 (26.6)	0	30 (100)	13 (46.4)	**<0.001**+
NSIP, *n* (%)	85 (42.5)	50 (55.5)	8 (26.6)	22 (100)	0	5 (17.8)	
Others, *n* (%)	29 (14.5)	5 (5.5)	14 (46.8)	0	0	10 (35.7)	

The distribution of key parameters across five ILD subtypes. Asterisks indicate levels of statistical significance, *p < 0.05; **p < 0.01; ***p < 0.001.

CTD-ILD, Connective tissue disease-associated interstitial lung disease; HP, Hypersensitivity pneumonitis; NSIP, Non-specific interstitial pneumonia; IPF, Idiopathic pulmonary fibrosis; BMI, Body mass index; FVC, Forced vital capacity; mMRC grade, modified Medical Research Council dyspnea grade; UIP, Usual interstitial pneumonia.

^@^ANOVA test; ^$^Chi-Square test; ^+^Fisher’s exact test; ^#^Kruskal-Wallis test.

The significant *p* values are presented in bold.

It was observed that IPF patients were older (66.97 ± 7.63 vs. 54.07 ± 15.97; *p* = 0.0001) than all other ILD subtypes. It was noted that, though women were predominant (137 patients, 68.5%) in patients with ILD, men formed the majority in IPF (56.7%) and ‘other ILDs’ (53.6%) subtypes. Fibrotic HP (10 patients, 33.3%) had higher occupational exposure to pollutants than CTD-ILD patients (7 patients, 7.7%). Comorbidity cases showed that 70 patients (35%) had systemic hypertension, 51 patients (25.5%) had type 2 diabetes mellitus, 13 patients (6.5%) had ischemic heart disease, 3 patients (1.5%) had a history of cerebrovascular accident, and 6 patients (3%) had chronic kidney disease. Patients with IPF had the highest percentage of at least one comorbidity (70%). It was noted that 35 patients (17.5%) had suffered from pulmonary tuberculosis in the past.

A higher percentage of predicted forced vital capacity (FVC) was noted in IPF patients as compared to fibrotic HP and idiopathic NSIP, respectively (64.70 ± 17.53 vs. 49.08 ± 16.26, *p* = 0.0052 and 49.38 ± 12.74, *p* = 0.0094). Among all patients with ILD, 150 patients (75%) presented with exertional breathlessness, while 118 patients (59%) had cough. IPF patients also had the shortest median duration of symptoms (7.5 months). A definite/probable usual interstitial pneumonia (UIP) pattern on HRCT chest was noted in 86 patients with ILD (43%), which was dominated by 35 CTD-ILD patients (38.8%). Among the 200 patients with ILD evaluated, ANA positivity was observed in 123 patients (61.5%). ANCA positivity was noted in 38 patients (19%) of the cohort, and anti-dsDNA autoantibodies were detected in only two patients (1%).

### Single nucleotide polymorphisms-*TOLLIP*

The *TOLLIP* rs3750920 SNP did not conform to the HWE in the ILD cohort (*p* = 0.015), while the control group did conform to the HWE (*p* = 0.21) (Table 2). Among all patients with ILD, 17.4% had the *TOLLIP* rs3750920 C/C genotype; 52.1% had the C/T genotype, and 30.5% had T/T genotype. Among IPF patients, 39.3% had T/T genotype. The T allele was more frequent in IPF as compared to controls (66.1% vs. 50.5%; OR = 1.91; 95% CI, 1.03–3.54; *p* = 0.038). The polymorphisms conformed to the HWE in ILD and controls for all other *TOLLIP* SNPs. Although the proportion of *TOLLIP* rs111521887_G was comparable between ILD and controls, it was more frequent in CTD-ILD as compared to controls (18.4% vs. 7.4%; OR = 2.83; 95% CI, 1.44–5.56; *p* = 0.002) (Table 3). For *TOLLIP* rs5743890, the frequency of the C/C genotype was most frequent in the healthy controls as compared to all patients with ILD and CTD-ILD (6.4% vs. 0.5 and 0%, respectively, *p* = 0.007 and 0.026, respectively), whereas the heterozygous genotype T/C was more frequent in CTD-ILD as compared to controls (5.9% vs. 2.1%, *p* = 0.026). The minor allele C of *TOLLIP* rs5743890 was more frequent in controls than ILD (7.5% vs. 2.9%; OR = 0.37; 95% CI, 0.0.16–0.83; *p* = 0.016) (Table 4). For *TOLLIP* rs5743894, genotype frequencies significantly differed in IPF patients compared to controls, with the heterozygous C/T genotype being more common in IPF (38.5% vs. 14.1%, *p* = 0.017). The *TOLLIP* rs5743894_C allele was more frequent in ILD than controls (14.5% vs. 8.1%, OR = 1.92; 95% CI, 1.09–3.416; *p* = 0.03) (Table 5). For *TOLLIP* rs5743854, the patients with the idiopathic NSIP subtype had more frequent G/G genotypes as compared to controls (29.4% vs. 2.9%, *p* = 0.001); therefore, the G allele was more frequent in idiopathic NSIP than controls (41.2% vs. 23.2%; OR = 2.32; 95% CI, 1.05–5.1; *p* = 0.033) (Table 6).

**Table 2:** Genotype and allele distribution of *TOLLIP* rs3750920.

*TOLLIP*. rs3750920	Genotypes			*p*-value	Alleles		*p*-value
	C/C (%)	C/T (%)	T/T (%)		C (%)	T (%)	
Healthy controls	23 (22.1)	57 (54.8)	24 (23.1)	0.32$	103 (49.5)	105 (50.5)	0.156$
All ILD	33 (17.4)	99 (52.1)	58 (30.5)		165 (43.4)	215 (56.6)	
CTD-ILD	13 (14.9)	49 (56.3)	25 (28.7)	0.38$	75 (43.1)	99 (56.9)	0.21$
Fibrotic HP	5 (17.8)	15 (53.6)	8 (28.6)	0.814$	25 (44.6)	31 (55.4)	0.54$
IPF	2 (7.1)	15 (53.6)	11 (39.3)	0.09$	19 (33.9)	37 (66.1)	**0.038**$
Idiopathic NSIP	4 (19.1)	9 (42.9)	8 (38.1)	0.35$	17 (53.1)	15 (46.9)	0.70$
Other ILDs	9 (34.6)	11 (42.3)	6 (23.1)	0.38$	29 (55.8)	23 (44.2)	0.44$

The genotype and allele distribution of the *TOLLIP* rs3750920 among healthy controls (*n* = 104) and patients with ILD subtypes (*n* = 190). Genotypic frequencies (C/C, C/T, T/T) and allelic frequencies (C, T) are shown as n (%). *p*-values indicate statistical significance for genotype and allele comparisons between controls and ILD groups, as well as among ILD subtypes.

CTD-ILD, Connective tissue disease-associated interstitial lung disease; HP, Hypersensitivity pneumonitis; NSIP, Non-specific interstitial pneumonia; IPF, Idiopathic pulmonary fibrosis.

^$^Chi-Square test.

The significant *p* values (<0.05) are presented in bold.

**Table 3:** Genotype and allele distribution of *TOLLIP* rs111521887.

*TOLLIP* rs111521887	Genotypes			*p*-value	Alleles		*p*-value
	C/C (%)	C/G (%)	G/G (%)		C (%)	G (%)	
Healthy controls	82 (86.3)	12 (12.6)	1 (1.1)	0.64+	176 (92.6)	14 (7.4)	0.625$
All ILD	141 (83.4)	27 (16)	1 (0.6)		309 (91.4)	29 (8.6)	
CTD-ILD	57 (78.1)	15 (20.6)	1 (1.3)	0.38+	129 (81.6)	29 (18.4)	**0.002**$
Fibrotic HP	22 (91.7)	2 (8.3)	0	0.78+	46 (95.8)	2 (4.2)	0.42$
IPF	24 (96)	1 (4)	0	0.44+	49(98)	1 (2)	0.16$
Idiopathic NSIP	16 (84.2)	3 (15.8)	0	0.76+	35 (92.1)	3 (7.9)	0.91$
Other ILDs	22 (78.6)	6 (21.4)	0	0.50+	48 (88.9)	6 (11.1)	0.37$

The genotype and allele distribution of the *TOLLIP* rs111521887 among healthy controls (*n* = 95) and patients with ILD subtypes (*n* = 169). Genotypic frequencies (C/C, C/G, G/G) and allelic frequencies (C, G) are shown as *n* (%). *p*-values indicate statistical significance for genotype and allele comparisons between controls and ILD groups, as well as among ILD subtypes.

CTD-ILD, Connective tissue disease-associated interstitial lung disease; HP, Hypersensitivity pneumonitis; NSIP, Non-specific interstitial pneumonia; IPF, Idiopathic pulmonary fibrosis.

^$^Chi-Square test; ^+^Fisher’s exact test.

The significant *p* values (<0.01) are presented in bold.

**Table 4:** Genotype and allele distribution of *TOLLIP* rs5743890.

*TOLLIP* rs5743890	Genotypes			*p*-value	Alleles		*p*-value
	C/C (%)	T/C (%)	T/T (%)		C (%)	T (%)	
Healthy Controls	6 (6.4)	2 (2.1)	86 (91.5)	**0.007**+	14 (7.5)	174 (92.5)	**0.016**$
All ILD	1 (0.5)	9 (4.7)	180 (94.7)		11 (2.9)	369 (97.1)	
CTD-ILD	0	5 (5.9)	80 (94.1)	**0.026**+	5 (2.9)	165 (97.1)	0.057$
Fibrotic HP	0	1 (3.6)	27 (96.4)	0.33+	1 (1.8)	55 (98.2)	0.12$
IPF	1 (3.6)	2 (7.1)	25 (89.3)	0.49+	4 (7.1)	52 (92.9)	0.93$
Idiopathic NSIP	0	0	21 (100)	0.72+	0	42 (100)	0.07+
Other ILDs	0	1 (3.6)	27 (96.4)	0.33+	1 (1.8)	55 (98.2)	0.12$

The genotype and allele distributions of *TOLLIP* rs5743890 among healthy controls (*n* = 94) and patients with ILD subtypes (*n* = 190). Genotypic frequencies (C/C, C/T, T/T) and allelic frequencies (C, T) are shown as *n* (%). *p*-values indicate statistical significance for genotype and allele comparisons between controls and ILD groups, as well as among ILD subtypes.

CTD-ILD, Connective tissue disease-associated interstitial lung disease; HP, Hypersensitivity pneumonitis; NSIP, Non-specific interstitial pneumonia; IPF, Idiopathic pulmonary fibrosis.

^$^Chi-Square test; ^+^Fisher’s exact test.

The significant *p* values (<0.05, <0.01) are presented in bold.

**Table 5:** Genotype and allele distribution of *TOLLIP* rs5743894.

*TOLLIP* rs5743894	Genotypes			*p*-value	Alleles		*p*-value
	C/C (%)	C/T (%)	T/T (%)		C (%)	T (%)	
Healthy Controls	1 (1.01)	14 (14.1)	84 (84.8)	0.059+	16 (8.1)	182 (91.9)	**0.03**$
All ILD	3 (1.7)	46 (25.6)	131 (72.7)		52 (14.5)	308 (85.5)	
CTD-ILD	1 (1.2)	22 (26.5)	60 (72.3)	0.057+	24 (14.5)	142 (85.5)	0.052$
Fibrotic HP	1 (3.8)	4 (15.4)	21 (80.8)	0.426+	6 (11.5)	46 (88.5)	0.43$
IPF	0	10 (38.5)	16 (61.5)	**0.017**+	10 (19.2)	42 (80.8)	**0.01**$
Idiopathic NSIP	0	5 (25)	15 (75)	0.428+	5 (12.5)	35 (87.5)	0.36$
Other ILDs	1 (4)	5 (20)	19 (76)	0.28+	7 (14)	43 (86)	0.19$

The genotype and allele distribution of *TOLLIP* rs5743894among healthy controls (*n* = 99) and patients with ILD subtypes (*n* = 180). Genotypic frequencies (C/C, C/T, T/T) and allelic frequencies (C, T) are shown as *n* (%). *p* values indicate statistical significance for genotype and allele comparisons between controls and ILD groups, as well as among ILD subtypes.

CTD-ILD, Connective tissue disease-associated interstitial lung disease; HP, Hypersensitivity pneumonitis; NSIP, Non-specific interstitial pneumonia; IPF, Idiopathic pulmonary fibrosis.

^$^Chi-Square test; ^+^Fisher’s exact test.

The significant *p* values (<0.05) are presented in bold.

**Table 6:** Genotype and allele distribution of *TOLLIP* rs5743854.

*TOLLIP* rs5743854	Genotypes			*p*-value	Alleles		*p*-value
	C/C (%)	C/G (%)	G/G (%)		C (%)	G (%)	
Healthy Controls	39 (56.5)	28 (40.6)	2 (2.9)	0.055$	106 (76.8)	32 (23.2)	0.57$
All ILD	86 (59.7)	42 (29.2)	16 (11.1)		214 (74.3)	74 (25.7)	
CTD-ILD	45 (68.2)	18 (27.3)	3 (4.5)	0.25$	108 (81.8)	24 (18.2)	0.31$
Fibrotic HP	11 (55)	6 (30)	3 (15)	0.10$	28 (70)	12 (30)	0.37$
IPF	10 (55.5)	5 (27.8)	3 (16.7)	0.06$	25 (69.4)	11 (30.6)	0.361$
Idiopathic NSIP	8 (47.1)	4 (23.5)	5 (29.4)	**0.001**$	20 (58.8)	14 (41.2)	**0.033**$
Other ILDs	12 (52.2)	9 (39.1)	2 (8.7)	0.49$	33 (71.7)	13 (28.3)	0.48$

The genotype and allele distribution of *TOLLIP* rs5743854 among healthy controls (n = 69) and patients with ILD subtypes (n = 144). Genotypic frequencies (C/C, C/G, G/G) and allelic frequencies (C, G) are shown as n (%). *p* values indicate statistical significance for genotype and allele comparisons between controls and ILD groups, as well as among ILD subtypes.

CTD-ILD, Connective tissue disease-associated interstitial lung disease; HP, Hypersensitivity pneumonitis; NSIP, Non-specific interstitial pneumonia; IPF, Idiopathic pulmonary fibrosis.

^$^Chi-Square test.

The significant *p* values (<0.05, <0.01) are presented in bold.

### Single nucleotide polymorphisms-*MUC5B*

The *MUC5B* minor T allele was more frequent in ILD than controls (19.6% vs. 5.8%; OR = 3.2; 95% CI, 1.58 to 6.3; *p* = 0.0008). The polymorphism conformed to the HWE in the controls (*p* = 0.4) but not in the ILD cohort (*p* = 0.0062). The ORs for ILD among heterozygous (G/T) and homozygous (T/T) subjects for the risk allele were 4.43 (95% CI, 2.23–8.9; *p* < 0.001) and 5.9 (95%CI, 0.88–66.22; *p* = 0.0008), respectively. The *MUC5B* minor T allele was more frequent in all the subtypes of ILD than in controls (Table 7).

**Table 7:** Genotype and allele distribution of *MUC5B* rs35705950.

*MUC5B* rs35705950	Genotypes			*p*-value	Alleles		*p*-value
	G/G (%)	G/T (%)	T/T (%)		G (%)	T (%)	
Healthy Controls	93 (89.4)	10 (9.6)	1 (1)	<0.001$	196 (94.2)	12 (5.8)	**0.0008**$
All ILD	126 (64.9)	60 (30.9)	8 (4.1)		312 (80.4)	76 (19.6)	
CTD-ILD	62 (70.5)	23 (26.1)	3 (3.4)	0.002+	147 (83.5)	29 (16.5)	**<0.001**$
Fibrotic HP	20 (71.4)	5 (17.8)	3 (10.7)	0.012+	45 (80.4)	11 (19.6)	**0.003**$
IPF	13 (44.8)	15 (51.7)	1 (3.4)	<0.001+	41 (70.7)	17 (29.3)	**<0.001**$
Idiopathic NSIP	12 (57.1)	9 (42.9)	0	0.001+	21 (70)	9 (30)	**0.001**$
Other ILDs	19 (67.9)	8 (28.6)	1 (3.5)	0.015+	46 (82.1)	10 (17.9)	**0.011**$

The genotype and allele distribution of the *MUC5B* promoter polymorphism rs35705950 among healthy controls (*n* = 104) and patients with ILD subtypes (*n* = 194). Genotypic frequencies (G/G, G/T, T/T) and allelic frequencies (G, T) are shown as n (%). p-values indicate statistical significance for genotype and allele comparisons between controls and ILD groups, as well as among ILD subtypes.

CTD-ILD, Connective tissue disease-associated interstitial lung disease; HP, Hypersensitivity pneumonitis; NSIP, Non-specific interstitial pneumonia; IPF, Idiopathic pulmonary fibrosis.

^$^Chi-Square test; ^+^Fisher’s exact test.

The significant *p* values (<0.05, <0.01, <0.001) are presented in bold.

### Haplotype identification and linkage disequilibrium

**Figure 1:**
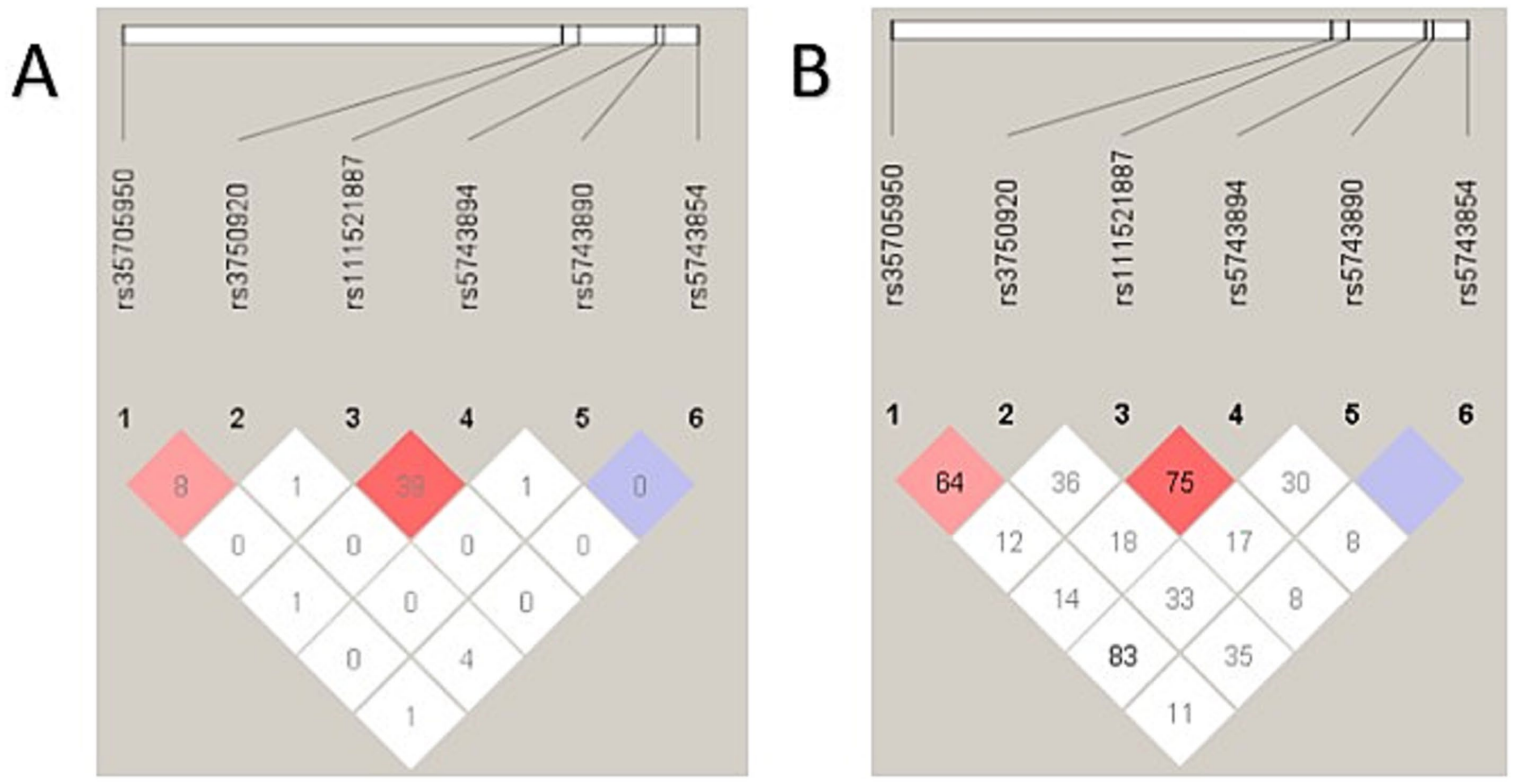
The squared Pearson’s correlation coefficient R^2^ **(A)**, and D prime **(B)** were used as a measure of LD between pairs of SNPs using Haploview. Low R^2^ values were consistently observed for all the SNP pairs. The SNPs rs111521887 and rs5743894 had a R^2^ value of 39 and a D’ value of 75, indicating no significant linkage. The 2 other pairs, with higher D’ values – rs35705950 and rs3750920, and the pair rs35705950 and rs5743890, show similar results with high D’ values but low R^2^. This pattern suggests that although recombination events between these loci may be limited (high D’), the allelic correlation is weak (low R^2^).

The R^2^ and D′ values were used to assess LD between pairs of *TOLLIP* SNPs and *MUC5B,* as both play important roles in ILD. As shown in Figure 1, R^2^ was low for all six pairs of SNPs in patients, indicating limited correlation. The *TOLLIP* SNPs rs111521887 and rs5743894 had an R^2^ value of 39 and a D′ value of 75, indicating no significant linkage. The 2 other pairs, with higher D′ values -*MUC5B* rs35705950 and *TOLLIP* rs3750920, and the pair *MUC5B* rs35705950 and *TOLLIP* rs5743890, showed similar results with high D′ values but low R^2^. For three SNP pairs, although D′ values were relatively high, the corresponding R^2^ values remained low, indicating limited correlation despite evidence of disequilibrium. Therefore, it was inferred that there were no meaningful haplotype blocks in the studied population.

### Distribution of serum cytokine levels in *TOLLIP* and *MUC5B* genotypes

Among all cytokines assessed across *TOLLIP* and *MUC5B* genotypes, statistically significant differences were observed only for IFN-*γ* levels. Serum IFN-γ levels varied significantly across *TOLLIP* rs5743854 genotypes (C/C: 2.16 pg/mL, C/G: 1.98 pg/mL, G/G: 1.82 pg/mL; *p* = 0.009) and *MUC5B* rs35705950 genotypes (G/G: 3.28 pg/mL, G/T: 2.08 pg/mL, T/T: 1.69 pg/mL; *p* = 0.034). Under the dominant model, *MUC5B* rs35705950 G/G homozygotes showed significantly higher IFN-γ levels compared to combined G/T + T/T genotypes (3.28 vs. 2.04 pg./mL; *p* = 0.033). No significant associations were found for IL-1β, TNF-*α*, or IL-6 with any of the genotypes tested (*p* > 0.05 for all) (Supplementary Tables S1, S2).

## Discussion

Interstitial lung diseases (ILDs) are a heterogeneous group of progressive fibro-inflammatory parenchymal disorders in which genetic predisposition plays a critical role alongside environmental triggers. Polymorphisms in *TOLLIP* and *MUC5B* have been widely implicated in ILD susceptibility and progression through their influence on innate immune signaling and mucosal epithelial integrity (4). Given the known differences in allele frequencies, linkage disequilibrium patterns, and ILD subtype distribution, no data are available on Indian patients with ILD. The present study addresses this gap by characterizing *TOLLIP* and *MUC5B* SNPs and their relationship with inflammatory biomarker profiles in patients with ILD from Western India.

For *TOLLIP* rs3750920, 17.4% of patients had the C/C genotype, 52.1% had C/T, and 30.5% had T/T genotype. Similarly, in the PANTHER trial, conducted in a Caucasian population, 28.1% of patients had the T/T genotype (11). In the present study, although the T allele frequency was not significantly higher when all patients with ILD were analyzed, the IPF subtype showed a significantly higher T allele frequency compared to controls. Similar findings have also been reported in other studies on IPF patients and in a study on IIP patients (2, 4, 11). The *TOLLIP* rs3750920_T allele is associated with response to NAC therapy as per the results of the PANTHER-IPF trial. This formed the basis for the PRECISIONS trial, one of the first to use a pharmacogenomic approach to treat ILDs (12, 25). As the PRECISIONS trial reported that IPF patients with the *TOLLIP* rs3750920_TT genotype can benefit from NAC therapy, the present study suggests that one-third of Indian patients with ILD carry this genotype and may benefit from NAC therapy. Isshiki et al. (26) found that *TOLLIP* rs3750920_T was less frequent among Japanese patients with ILD than in Western populations, in which 6% of non-IPF fibrosing patients with ILD and 7% of IPF patients had the T/T genotype. Our study suggested that Indian patients with ILD conform to Caucasian allele distribution in *TOLLIP* rs3750920, a finding with potential pharmacogenomic implications. *TOLLIP* rs111521887_G was more frequent in CTD-ILD patients; however, there was no statistically significant difference when all ILDs were compared with controls in this study. Li et al. (4) reported that the minor allele G was more frequent in patients with ILD, with five studies of IPF patients and one study on RA-ILD involved. Mota et al. (14) reported higher minor allele G frequency in fibrotic HP. In the present study, for *TOLLIP* rs111521887, the minor allele frequency was lower in IPF patients than in controls. This may be due to the smaller sample size of IPF patients.

In the PANTHER trial, 16.1% patients were heterozygous (T/C) for *TOLLIP* rs5743890, which was higher than 4.7% heterozygous (T/C) reported in this study (11). The present study also highlighted that for *TOLLIP* rs5743890, minor allele C was more frequent in controls than in patients with ILD. This finding was consistent with the meta-analysis by Li et al. (4), in which 18.9% controls and 11.4% patients with ILD were *TOLLIP* rs5743890 minor-allele C carriers. This meta-analysis included 8 studies, of which 6 included only IPF patients. In the remaining two studies that included RA-ILD and fibrotic HP patients, the minor allele C was more frequent in subtypes of ILD. In the present study, although subtype analysis did not reach statistical significance, the minor allele C was less common in ILD. This further strengthened the possibility that *TOLLIP* rs5743890_C is protective against ILD, consistent with the results of the genome-wide association study (GWAS) reported by Noth et al. (2) on IPF patients. A meta-analysis by Li et al. (4) also suggested that patients with ILD with the minor allele C had the worst prognosis and survival. Mota et al. (14) reported a higher frequency of *TOLLIP* rs5743894_C in fibrotic HP patients (23.2%) than controls (12.9%). Similarly, *TOLLIP* rs5743894_C was more frequent in patients with ILD (14.5%) than in controls (8.1%) in the present study. In the PANTHER trial of 154 IPF patients, 40.9% were heterozygotes (T/C) and 8.4% were homozygous(C/C) for the minor allele C at *TOLLIP* rs5743894. In this study, although the minor allele C was more frequent across all subtypes of ILD than in controls, in IPF, this difference was statistically significant (11, 13). *TOLLIP* rs5743854_G was more frequent in idiopathic NSIP in the present study; Mota et al. (14) reported no statistically significant difference in allele distribution for this SNP in fibrotic HP patients. To the best of our knowledge, this was the first report on the association of *TOLLIP* rs5743854_ with idiopathic NSIP. *TOLLIP* is a versatile protein, postulated to regulate inflammation. *TOLLIP* expression is associated with the *TOLLIP* genotype. Patients with ILD and rs5743890_C had 20% lower *TOLLIP* expression; it was 40% less in those with rs111521887_G, and 50% lower in those with rs5743894_G. *TOLLIP* deficiency has been reported to be associated with susceptibility to IPF (3, 4). Although no meaningful haplotype blocks were identified in the present study, Mota et al. (14) reported that haplotypes formed by *TOLLIP* rs3750920, rs111521887, and rs5743894 and *MUC5B* rs37505950 were associated with susceptibility to fibrotic HP and that the G-T-G-C haplotype predicted decreased survival. Schroder et al. (15) had reported that the haplotype T-C formed by *TOLLIP* rs3750920 and rs5743890 predicted progression in SSc-ILD patients.

Genotype–phenotype correlation was reported in fibrotic HP patients by Katayanagi et al. (27), who demonstrated that serum levels of IL-1β, TNF-*α*, IFN-*γ*, and IL-6 were associated with *TOLLIP* genotypes. In this study, IFN-γ levels were higher in the *TOLLIP* rs5743854, C/C genotype and *MUC5B* rs35705950 G/G genotype than in other heterozygous and recessive genotypes of both SNPs. IFN-γ is a pro-inflammatory cytokine produced by T cells and NK cells that activates macrophages and sustains lung inflammation in ILD. *TOLLIP* is a negative regulator of TLR signaling and limits downstream cytokine production, including IFN-γ. The C/C genotype at *TOLLIP* rs5743854 has been associated with a relatively reduced *TOLLIP* expression, leading to less suppression of TLR signaling and consequently higher IFN-γ levels (28, 29). For *MUC5B* rs35705950, the minor T allele is a gain-of-function variant that increases *MUC5B* expression, resulting in a dampened Th1 response and lowering IFN-γ levels (5). Conversely, G/G individuals with baseline *MUC5B* expression maintain intact epithelial immune surveillance, supporting higher IFN-γ production. Further studies on larger populations are needed to determine their significance. Given that this was a cross-sectional, single-center study, the sample size for each ILD subtype was limited. Furthermore, patients categorized as the “Other ILDs” subtype included a heterogeneous group of diseases. Still, this remains the first comprehensive study on *TOLLIP and MUC5B* genotypes in Indian subtypes of ILD. Additionally, the distribution of cytokine levels across the heterozygous, dominant, and recessive homozygous genotypes of *TOLLIP* and *MUC5B* SNPs provided further insight regarding genotype–phenotype associations.

## Conclusion

This study provided comprehensive insights into the genotype and allele frequencies of *TOLLIP* and *MUC5B* SNPs across Indian subtypes of ILD, with minor alleles more frequent in all patients with ILD and their respective ILD subtypes for *TOLLIP* rs3750920, rs11152187, rs5743894, and rs5743854. However, the minor allele for *TOLLIP* rs5743890 was found to be protective for ILD. Higher IFN-γ levels among homozygous dominant genotypes of *TOLLIP* and *MUC5B* raised the possibility that these patients may represent a subgroup with a distinct immunological endotype, warranting a longitudinal follow-up to determine whether this immune profile confers differential treatment responsiveness, particularly to immunomodulatory therapies.

## Data Availability

The raw data supporting the conclusions of this article will be made available by the authors, without undue reservation.
