# Supplementary material for: TOLLIP and MUC5B single nucleotide polymorphisms among interstitial lung disease patients from Western India
Source: Front Med (Lausanne). 2026 Apr 14;13:1806788. doi: 10.3389/fmed.2026.1806788 (PMC13121113; doi:10.3389/fmed.2026.1806788)
Supplement: Supplementary file 1 [file Data_Sheet_1.docx]

***TOLLIP* and *MUC5B* single nucleotide polymorphisms among interstitial lung disease patients from Western India**

Tanya Athavale^1^, Amita Athavale^1^, Trisha Samant^2^, Namrata Neman^2^, Ridi Khatri^2^, Pooja Jaiswal^2^, Kunal Dabholkar^2^, Somprakash Dhangar^2^, Anshu Priya^1^, Manisha Madkaikar^3^, Vandana Pradhan^2^

**Online Supplementary Data**

**Table S1**: Distribution of serum cytokine levels (pg/ml) in *TOLLIP* and *MUC5B* genotypes

| SNPs | Cytokines levels in (pg/ml) | Genotypes | | | p value |
| --- | --- | --- | --- | --- | --- |
| *TOLLIP* rs3750920 |  | C/C | C/T | T/T |  |
|  | IL-1β  TNF-α  IFN-γ  IL-6 | 2.58  (1.26-4.44)  3.20  (1.59-15.55)  5.15  (3.79-22.04)  51.49  (24.35-370.70) | 2.40  (1.76-6.00)  2.20  (1.64-13.08)  5.20  (3.82-7.87)  96.90  (40.68-388.90) | 3.79  (2.07-5.98)  2.19  (1.52-4.52)  4.81  (3.82-6.64)  72.74  (43.80-323.50) | 0.562  0.741  0.680  0.328 |
|  |  | C/C | C/G | G/G |  |
| *TOLLIP* rs11152187 | IL-1β  TNF-α  IFN-γ  IL-6 | 2.72  (1.73-5.94)  2.19 (1.52-111.38)  4.98  (3.81-7.08)  100.30  (40.41-401.60) | 2.07  (1.61-3.33)  1.73  (1.58-3.10)  5.38  (3.78-6.74)  43.15  (26.93-145.50) | ----  ----  ----  ---- | 0.188  0.223  0.81  0.059 |
|  |  | T/T | T/C | C/C |  |
| *TOLLIP* rs5743890 | IL-1β  TNF-α  IFN-γ  IL-6 | 2.75  (1.68-5.98)  2.25  (1.61-12.08)  5.03  (3.82-7.13)  83.80  (37.57-359.50) | 2.12  (1.90-3.21)  1.60  (1.24-4.63)  5.78  (3.78-6.96)  98.77  (27.62-414.20) | ----  ----  ----  ---- | 0.453  0.191  0.97  0.666 |
|  |  | T/T | T/C | C/C |  |
| *TOLLIP* rs5743894 | IL-1β  TNF-α  IFN-γ  IL-6 | 2.75  (1.66-5.97)  2.80  (1.64-12.52)  5.06  (3.83-7.18)  125.40  (40.68-398.50) | 2.72  (1.72-5.83)  2.03  (1.61-5.70)  5.04  (3.84-6.74)  60.87  (29.77-188.70) | 2.70  (1.84-3.57)  1.52  (1.23-1.82)  3.01  (2.24-3.78)  33.23  (23.75-42.71) | 0.926  0.345  0.14  0.095 |
|  |  | C/C | C/G | G/G |  |
| *TOLLIP* rs5743854 | IL-1β  TNF-α  IFN-γ  IL-6 | 2.36  (1.45-5.18)  2.16  (1.51-14.81)  5.07  (3.82-8.74)  88.06  (40.53-313.10) | 2.66  (1.88-5.52)  1.98  (1.60-6.19)  5.01  (3.84-7.03)  70.90  (30.03-392.00) | 2.03  (1.63-9.25)  1.82  (1.23-3.26)  3.78  (3.08-5.03)  43.15  (23.75-681.20) | 0.529  0.346  **0.009 ****  0.757 |
|  |  | G/G | G/T | T/T |  |
| *MUC5B* rs35705950 | IL-1β  TNF-α  IFN-γ  IL-6 | 2.30  (1.60-5.57)  3.28  (1.61-14.68)  6.41  (3.85-7.74)  90.72  (35.30-420.40) | 3.02  (1.93-6.16)  2.08  (1.60-4.05)  4.98  (3.82-6.46)  74.80  (39.07-276.10) | 3.06  (2.55-4.80)  1.69  (1.23-4.89)  3.81  (3.78-4.81)  42.71  (13.65-867.20) | 0.286  0.211  **0.034***  0.645 |

**Table S1** represents the distribution of serum cytokine levels (pg/ml) in *TOLLIP* and *MUC5B* genotypes. Values are represented as median (Q1-Q3). Statistical comparisons were performed using Kruskal-Wallis test for multiple comparisons followed by pair wise post hoc analysis using Dunn’s test with Bonferroni correction. Mann Whitney U test was used for comparison between two groups. A p value < 0.05 was considered statistically significant (marked with *) and presented in bold

**Table S2** : Comparison of serum cytokine levels (pg/ml) between homozygous dominant genotype and combined genotypes (Heterozygous genotype + homozygous recessive genotypes) of *TOLLIP* and *MUC5B* SNPs

| SNPs | Cytokines levels in (pg/ml) | Combined Genotypes | | p value |
| --- | --- | --- | --- | --- |
| *TOLLIP* rs3750920 |  | C/C | C/T + T/T |  |
|  | IL-1β  TNF-α  IFN-γ  IL-6 | 2.58  (1.26-4.44)  3.20  (1.59-15.55)  5.15  (3.79-22.04)  51.49  (24.35-370.70) | 2.75  (1.84-6.00)  2.19  (1.61-6.71)  5.03  (3.82-7.07)  89.43  (42.71-359.50) | 0.524  0.644  0.771  0.145 |
|  |  | T/T | T/C + C/C |  |
| *TOLLIP* rs5743894 | IL-1β  TNF-α  IFN-γ  IL-6 | 2.75  (1.66-5.97)  2.80  (1.64-12.52)  5.06  (3.83-7.18)  125.40  (40.68-398.50) | 2.72  (1.75-5.47)  1.96  (1.61-4.24)  4.98  (3.82-6.65)  57.59  (29.34-173.20) | 0.809  0.392  0.739  0.062 |
|  |  | C/C | C/G + G/G |  |
| *TOLLIP* rs5743854 | IL-1β  TNF-α  IFN-γ  IL-6 | 2.36  (1.45-5.18)  2.16  (1.51-14.81)  5.07  (3.82-8.74)  88.06  (40.53-313.10) | 2.55  (1.80- 5.57)  1.92  (1.52-5.24)  4.10  (3.77-6.52)  64.95  (28.98-395.20) | 0.298  0.268  0.053  0.459 |
|  |  | G/G | G/T + T/T |  |
| *MUC5B* rs35705950 | IL-1β  TNF-α  IFN-γ  IL-6 | 2.30  (1.60-5.57)  3.28  (1.61-14.68)  6.41  (3.85-7.74)  90.72  (35.30-420.40) | 2.06  (2.05-6.07)  2.04  (1.56-4.05)  4.81  93.79-6.12)  72.74  (37.22-286-80) | 0.124  0.129  **0.033***  0.404 |

**Table S2** represents the comparison of serum cytokine levels (pg/ml) between homozygous dominant genotype and combined genotypes (Heterozygous genotype + homozygous recessive genotypes) of *TOLLIP* and *MUC5B* SNPs. Values are represented as median (Q1-Q3). Statistical comparisons were performed using Mann Whitney U test. A p value < 0.05 was considered statistically significant (marked with *) and presented in bold
